# Toxigenicity and phylogeny of *Aspergillus* section *Flavi *in poultry feed in Iran 

**DOI:** 10.18502/cmm.6.1.2504

**Published:** 2020

**Authors:** Seyed Soheil Ghaemmaghami, Nasrin Pashootan, Mehdi Razzaghi-Abyaneh

**Affiliations:** 1 Feed Hygienist, Institute of Agricultural Education and Extension, Agricultural Research, Education and Extension Organization (AREEO), Tehran, Iran; 2 Department of Mycology, Pasteur Institute of Iran, Tehran, Iran

**Keywords:** Aflatoxigenic strains, Aflatoxin B_1_, Aspergillus section Flavi, Corn, Poultry feed, Phylogenetic tree

## Abstract

**Background and Purpose:**

This study was conducted to evaluate the presence of aflatoxigenic strains and level of aflatoxin in poultry feed. Aflatoxigenic strains were investigated in corn and soybean meal as the ingredients of poultry feed, as well as in two types of commercial feed, namely pellet and mash. The gene sequencing was performed to identify the species of *Aspergillus* section *Flavi.*

**Materials and Methods:**

All samples were randomly collected from feed storage silos located in Iran in 2018. The samples were cultured on specialized media for 2 weeks at 28ºC. Identification of *Aspergillus* section *Flavi *isolates was based on macro- and microscopic morphological criteria and molecular analysis. The thin-layer chromatography (TLC) was applied to confirm the aflatoxigenic isolates. In addition, the level of aflatoxin B_1_ (AFB_1_) produced by these isolates was determined by high-performance liquid chromatography. The strains were subjected to sequence analysis, and Bt2 PCR products were purified by the QIAquick PCR purification kit. At the final stage, the phylogenetic tree was built.

**Results:**

Among 54 isolates identified as *Aspergillus* section *Flavi,* 20 (37%) isolates were found to produce aflatoxin at a range of 11.28±1.18 to 2239.92±92.26 µg/g fungal dry weight. The aflatoxigenic isolates had the frequencies of 45%, 40%, 10%, and 5% in the corn, pellet, soybean meal, and mash samples, respectively. Furthermore, the mean concentrations of AFB_1_ were significantly higher in the corn samples (707.04±39.05) than that of other poultry feed samples (*P<0.05*). A total of 34 (63%) isolates were detected as non-aflatoxigenic on the yeast extract-sucrose broth in TLC analysis. The toxigenic isolates produced the highest (2232.62±55.49) and lowest (11.28±1.18) levels of AFB_1_ in the corn samples, compared to other feedstuffs. Furthermore, the mean level of AFB_1_ in mash product was 554.09±10.36 µg/g, compared to a mean level of 229.22±11.09 µg/g in pellets. The isolates were randomly selected, sequenced, and then analyzed. Subsequently, the phylogenetic tree of *Aspergillus* section *Flavi* was plotted.

**Conclusion:**

The process of converting raw ingredients to compound poultry feed is more hazardous when there is not enough time and temperature provided to eliminate aflatoxigenic isolates. Therefore, *Aspergillus *section *Flavi* in poultry feed can pose a threat to the poultry industry and poultry products, thereby affecting the health status of humans. Unprocessed/processed materials, such as corns and pelleted feed, need further monitoring, especially when conditions are not optimal for destroying the fungus.

## Introduction

Mycotoxin contamination of feedstuffs, especially poultry feed, is a great concern for animal health. According to the possible carry-over of each toxin, feed contamination can also pose a hazard to animal food origin, thereby leading to mycotoxin intake in humans [1-5]. *Aspergillus flavus* is a dominant pathogen in animal feed at poultry farms that can be divided into toxigenic and non-toxigenic strains. Aflatoxins (AFs) are important fungal toxic compounds, produced by an expanding list of closely related fungi, mainly *A. flavus* and *A. parasiticus* [6].The growth of *A. flavus* on raw materials and mixed poultry feed may induce suppressing effects on the immune system and consequently increase the rate of mortality in poultry farms. The thermoresistance of *A. flavus* may account for their potential to produce AFs in poultry feed [7, 8]. Poultry is one of the most sensitive animal groups to aflatoxicosis caused by AFs. There is evidence regarding the presence of toxicogenic fungi in poultry feed (mash and pellet) in Iran [9]. Aflatoxin B_1 _(AFB_1_) is considered one of the most important mycotoxins given its extreme toxicity and widespread presence in staple food and feed [10], as well as the possibility of its transition from animal feed to human food chain. According to the International Agency for Research on Cancer, AF has been introduced as a human liver carcinogen type A [11]. Aflatoxin contamination is a major concern for human health as AFB_1_ can be passed from poultry products, such as meat and eggs, to humans, even at a very low level [12, 13]. Different levels of this toxin can be found in maize and other poultry feed [14]. Morphological methods present invalid results for the identification of the species of *Aspergillus* section *Flavi* because of both intra-species similarities and differences [15]. On the other hand, the polymerase chain reaction (PCR)-based molecular assessment of microorganisms offers the specificity and sensitivity suitable for early detection [16].

The compatibility of β-tubulin gene sequencing for the identification of the species boundaries among *Aspergillus *section* Flavi* strains has been investigated in a study [17]. However, the present study was conducted to evaluate the presence of *Aspergillus* section *Flavi* in poultry feed can produce AFB_1. _This study was also targeted toward investigating the ability of *Aspergillus* section *Flavi* to produce different amounts of AFB_1_ using high-performance liquid chromatography (HPLC), as well as designing the phylogenetic tree of *Aspergillus *section *Flavi* in poultry feed in Iran.

## Materials and Methods

***Sampling of poultry feed***

This study was conducted on 85 samples of poultry feed randomly selected from storage of feed production companies licensed by the Iran Veterinary Organization in 2018. The companies were selected based on poultry feed production in provinces. The locations chosen for the study had the highest production potential in the Iranian poultry industry. Sampling was performed based on the type of feedstuff [18, 19]. The samples included corn (*Zea mays*) and soybean (*Glycine max*) as they are important raw materials (70%) used for producing poultry feed in Iran. The samples were also of two types of mash and pelleted feed. Mash is the simplest solid form of feed that involves grinding and mixing all raw materials at correct proportions to meet the nutritional requirements of animals before pelleting. Pelleted feed is agglomerated by compacting and forcing through die device by any mechanical process. The major origin ingredients of soybean and livestock corn in Iran are imported. In this research, the raw materials of the samples under study were imported from South American countries.

Sampling was performed randomly to provide an equal chance of selection from storage silos. The sampling was performed at several points (1 kg/point to 10 kg/point) to achieve an equal chance of selecting bulk feed; then, they were placed in clean zipper storage bags. The sub-samples selected for examination (500 g) were placed into sterile plastic bags and then transferred to the Mycology Laboratory at the Pasture Institute of Iran. The samples were stored at 4ºC before use.

***Isolation and identification***

To isolate the fungi, the sub-samples of each feed were separately cultured on selective isolation media using the spread plate method[20]. At first, the samples were homogenized and then a part of each sample, weighing 20 g, was mixed with 180 ml saline solution (0.85% sodium chloride) and 0.05% Tween. The resultant mixture was shaken for 30 min. In the next stage, 0.5, 0.75, and 1 ml of dilution were transferred and cultured on Sabouraud dextrose agar (SDA, E. Merck, Germany) plates containing 0.005% chloramphenicol (E. Merck, Germany), Dichloran rose bengal chloramphenicol agar (DRCA, Thermo Scientific™ Oxoid™, UK), and *Aspergillus flavus* and *A.* *parasiticus* agar (AFPA, Thermo Scientific™ Oxoid™, UK). 

The cultures were incubated at 28ºC for at least 2 weeks. In general, colonies on AFPA were yellow-orange in reverse color as a specific identification criterion. The species were also isolated by transferring the colonies grown on AFPA and/or DRCA to Potato dextrose agar plates. Additionally, Czapek doxagar was used to observe the morphological traits of the *Aspergillus *section* Flavi *colonies. Final identification of *Aspergillus *was performed based on a combination of macroscopic and microscope characteristics exhibited by species criteria [20, 21]. 

***Aflatoxin-producing capability***

The AF-producing ability of *A. flavus* was determined using the yeast extract-sucrose (YES) broth. In addition, the isolates were cultured on 2% yeast extract and 18% sucrose medium according to a study performed by Razzaghi-Abyaneh et al [22]. 

The medium was divided into 5-ml aliquots, transferred to Erlenmeyer flasks with a capacity of 25 ml, and sterilized by autoclaving at 121ºC for 15 min. The cultures were incubated at 28ºC for 96 h in static conditions after inoculation with 1×10^6 ^fungal conidia/ml standard. The total contents of each well, including the culture media and fungal biomass, were filtered through a thin layer of cheesecloth and then thoroughly washed with distilled water. A known weight of mycelia was placed in a stainless steel container and allowed to dry at 80ºC until obtaining a constant weight. Subsequently, the net dry weight of mycelia was determined.

***Qualification of aflatoxin B***_1_ ***by thin-layer chromatography***

Three replicates of each isolate culture were screened by thin-layer chromatography (TLC) on silica gel plates. The AFB_1_ standard (Sigma-Aldrich Chemical Co. St. Louis, Mo. the USA) and TLC silica gel 60 F_254_ plates (E. Merck, Germany) were used to detect AFs. All other solvents and reagents of analytical grade were supplied from the E. Merck Company (Germany). The AFs were visualized under ultraviolet (UV) light (365 nm) and photographed with a TLC scanner CAMAG Reprostar 3 (CAMAG, Switzerland). The AFB_1_ was quantified in all samples extracted from YES broth with chloroform using a separator funnel. In the next stage, 25 g of each sub-sample was extracted with 100 ml (90:10, v/v) in a blender jar. 

The chloroform extracts were concentrated in a rotary evaporator (EYELA N-1000, Japan) to near dryness and analyzed using TLC on 20×20-cm silica gel 60F_254_ plates (E. Merck, Darmstadt, Germany). The TLC plates were developed using chloroform: methanol (98:2, v/v) as the mobile phase. The AFs B_1_ and/or B_2_ were observed under UV light as blue spots.

***Quantification of aflatoxin B***_1_ ***by high-performance liquid chromatography***

The AFB_1_ was quantified in positive samples and measured by HPLC [23]. To this end, 50 µl of each sample (culture filtrate) was injected into the HPLC column (TSKgel ODS-80TS; 4.6 mm ID × 15.0 cm, TOSOH BIOSCIENCE, JAPAN) and eluted at a flow rate of 1 ml/min by water: acetonitrile: methanol (60:25:15 v/v/v). The amount of AFB_1_ was calculated at a wavelength of 365 nm by comparing the under-curve area of unknown samples with standards treated in the same manner. The retention time of AFB_1_ was 10.0 min. 

***DNA extraction***

Fungal strains were cultured on SDA (Merck, Germany) and incubated at 27°C for 4 days. Briefly, the up mycelial mass was flash-frozen in liquid nitrogen, and a soft powder was obtained in a porcelain mortar. The mycelia powder was added to a 300-μL DNA extraction buffer (containing 100 mM Tris-HCl, 10 mM EDTA, 0.5% w/v SDS, and 100 mM NaCl at a pH of 7.5), as well as 100 μL sodium acetate (pH=5.2) and kept at -20°C for 20 min. Subsequently, 300 μL of phenol:chloroform:isoamyl alcohol (25:24:1) was vortexed for 2 min and centrifuged at 7,000 rpm for 5 min. The supernatant was transferred to a new tube and added 400 μL chloroform. DNA was precipitated with an equal volume of 2-propanol, kept at -20°C for 20 min, and centrifuged at 12,000 rpm for 5 min. The precipitate was washed with 500 μL of 70% ethanol, centrifuged at 12,000 rpm for 3 min, and dried. Eventually, the DNA was suspended in 30 μL of sterile distilled water.

***Primers and amplification conditions***

Out of 20 strains, 5 species were used in sequence analysis. The primer used in this study was β tubulin (Bt2a) (5-GGTAACCAAATCGGTGCTGCTTTC-3) and Bt2b (5-ACCCTCAGTGTAGTGACCCTTGGC-3). The PCR reactions were prepared in the final volumes of 25 μL, containing 12.5 μL premix (Ampliqon, Denmark), 1 μL DNA template, and 1 μM of each forward and reverse primers, and the final volume was obtained with distilled water. The PCR cycling conditions included 5 min of initial pre-incubation at 94°C, followed by 35 cycles consisting of denaturation at 94°C for 45 sec, annealing at 60°C for 45 sec, and extension at 72°C for 1 min, with a final extension at 72°C for 5 min. Furthermore, 5 μL of the PCR products were electrophoresed on the 1% agarose gel in TBE buffer.

***Sequencing***

The Bt2 PCR products were purified using a QIAquick purification kit (Qiagen) and an ABI PRISM BigDye Terminator Cycle Sequencing Ready Reaction kit (Applied Biosystems, USA). These products were sequenced using an automated DNA Sequencer (ABI Prism 3730 Genetic Analyzer; Applied Biosystems) and edited with MEGA (version 7) [24] and Geneious software (http://www.geneious.com). The data were contrasted in GenBank using the BLASTn algorithm. Additionally, strain information was evaluated using the reference strains of NCBI (http://www.ncbi.nlm.nih.gov).

***Sequence analysis***

The phylogenetic tree was built using MEGA7 software [24], and the tree topology was evaluated visually for the congruence of species-rank clades with the following conditions: Statistical method was maximum likelihood, the test of phylogeny was neighbor-joining method with 1,000 replication, substitution model was Tamura-Nei, and there were uniform rates among sites.

***Statistical analysis***

The results were analyzed using one-way ANOVA with Tukey post hoc test. A *p-*value less than 0.05 were considered statistically significant. 

## Results

***Incidence of aflatoxigenic fungi in poultry feed***

**Figure 1.**
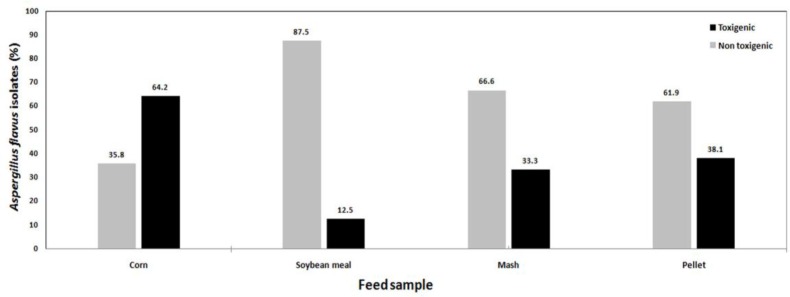
Frequency of *Aspergillus* section *Flavi* isolates (toxigenic/ non-toxigenic) in different feedstuffs

A total of 54 fungal isolates were collected out of 85 poultry feed samples under examination*.* Aspergillus* flavus* was the most common isolate .The investigation of the aflatoxigenic isolates recovered from poultry feed and their ingredients were accomplished by TLC (Table 1). Aflatoxigenic isolates (n=20) had the frequencies of 45%, 40%, 10%, and 5% in the corn, pellet, soybean meal, and mash samples, respectively. The corn samples had the highest proportion of aflatoxigenic isolates (9/14; 64.2%), followed by pelleted (8/21; 38.1%) and mash feed (1/3; 33.3%), with the soybean meal having the lowest level (2/16; 12.5%). Depicts the rate of isolates (toxigenic/non-toxigenic) in different feedstuffs (Figure 1). The rate of non-toxigenic isolates was higher than that of toxigenic isolates. Based on TLC analysis, out of 54 isolates, 34 (63%) cases were non-aflatoxigenic, while 20 (37%) cases were able to produce AFs on YES broth.

**Table 1:** Aflatoxigenic isolates^1^ obtained from poultry feed and their ingredients by thin-layer chromatography

**Isolates (n=54) ** ***Aspergillus *** **section** *** Flavi ***	**Ingredients (n=30)**				**Poultry feed (n=24)**			
	**Corn (n=14)**		**Soybean (n=16)**		**Mash (n=3)**		**Pellet (n=21)**	
	**No (%** ^2^ **)**	**No (%** ^3^ **)**	**No (%** ^2^ **)**	**No (%** ^3^ **)**	**No (%** ^2^ **)**	**No (%** ^3^ **)**	**No (%** ^2^ **)**	**No (%** ^3^ **)**
Aflatoxigenic (20)	9 (16.6)	9 (45)	2 (3.7)	2 (10)	1 (1.8)	1 (5)	8 (14.8)	8 (40)
Non-toxigenic (34)	5 (9.25)	5 (25)	14 (25.9)	14 (70)	2 (3.70)	2 (10)	13 (24.07)	13 (60)

^1^Aflatoxin production was identified as blue fluorescence on silica gel 60F_254_ thin-layer chromatography plates under ultraviolet (365 nm) light.

^2 ^Frequency of isolates based on total* Aspergillus *section* Flavi* isolates (n=54)

^3^Frequency of isolates based on total aflatoxigenic isolates (n=20)

**Table 2:** Results (mean ± SD) of high-performance liquid chromatography regarding the production of aflatoxin B_1_ by toxigenic *Aspergillus flavus* isolates on yeast extract-sucrose broth

**AFB**_1_ ** mean (µg/g)**	**AFB1 concentration (µg/g dry weight)**	**Fungal dry weight (mg)**	**Positive on TLC** **test**	**Feed**
707.04±39.05	2232.62±55.49	112.4±18.0	*A. flavus 101*	Corn
	1142.94±67.07	88.2±12.1	*A. flavus 105*	
	467.45±105.74	85.2±15.4	*A. flavus 117*	
	101.37±7.84	100.1±9.8	*A. flavus 109*	
	11.28±1.18	103.7±8.9	*A. flavus 111*	
	64.32±12.51	78.9±12.5	*A. flavus 104*	
	90.63±7.02	83.9±9.7	*A. flavus 112*	
	2239.92±92.26	109.2±12.9	*A. flavus 115*	
	12.89±2.39	90.8±13.6	*A. flavus 103*	
94.34±6.7	97.01±7.02	95.1±10.8	*A. flavus 102*	Soybean
	91.68±6.43	115.3±12.4	*A. flavus 113*	
554.09±10.36	554.9±10.36	91.1±11.7	*A. flavus 107*	Mash
229.22±11.09	185.24±6.39	96.9±8.7	*A. flavus 118*	Pellet feed
	167.04±5.43	106.3±9.6	*A. flavus 108*	
	193.97±6.75	88.1±10.6	*A. flavus 114*	
	179.03±10.22	100.5±12.9	*A. flavus 116*	
	288.07±10.93	92.1±7.3	*A. flavus 119*	
	120.72±11.14	111.82±7.1	*A. flavus 121*	
	296.7±18.12	111.82±7.1	*A. flavus 110*	
	402.98±19.72	111.82±7.1	*A. flavus 120*	

***Quantification of aflatoxin by toxigenic isolates***

The results of the HPLC analysis regarding the mean level of AFB_1_ produced by toxigenic isolates on YES broth are presented in Table 2. The mean level of AFB_1_ produced in pellet feed had the range of 120.72±11.14 to 402.98±19.72 µg/g. This mean value was 554.09±10.36 µg/g fungal dry weight in the mash feed. However, regarding the poultry ingredients, the corn samples were contaminated with an AFB_1_ range of 11.28±1.18 to 2239.92±92.26 µg/g fungal dry weight, and soybean meal had the mean AFB_1_ contamination range of 91.68±6.43 to 554.9±10.36 µg/g.

These results indicated that the strongest toxigenic strain was found in corn (2239.92±92.26) and the weakest contribution of AFB_1 _was obtained also from corn (11.28±1.18) µg/g. The mean concentration of AFB_1 _was at the highest level (707.04 ±39.05) in the corn samples. This mean value was significantly different (*P<0.05*) from the mean AFB_1 _found in other feedstuffs. On the other hand, the soybean meal samples had the lowest mean concentration of AFB_1_ (94.34±6.7) among all samples.

**Figure 2:**
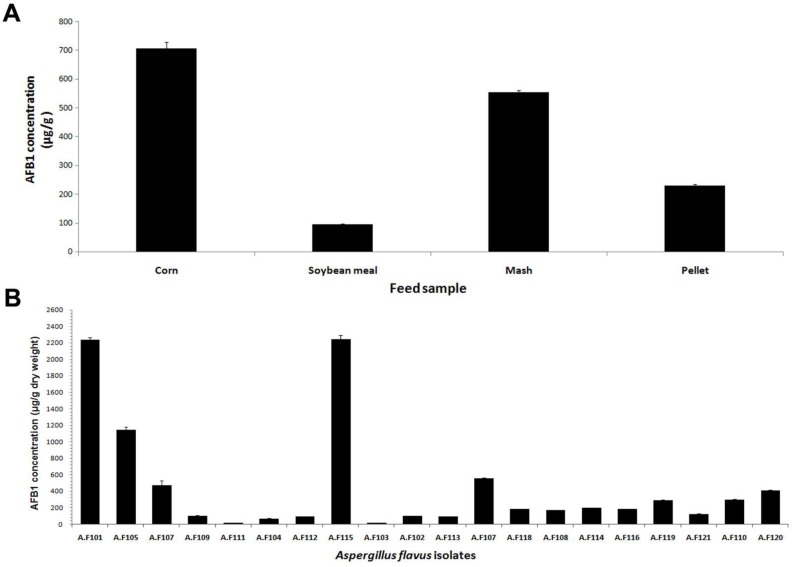
A) Aflatoxin B1 concentration in poultry feed samples and their ingredients B) AFB1 production by toxigenic A. flavus isolates

Figure 2a displays the mean levels of AFB_1_ based on pool SD (µg/g) dry weight and their concentrations in all feed samples (corn>mash>pellet>soybean). The examination of the quantity of the AFB_1_ produced in the feedstuffs inoculated with toxigenic isolates revealed that 70% of the isolates produced AFB_1_ in quantities exceeding 100.00 µg/g (pellets [35%], corn [30%], mash [5%]( and soybean meal isolate under 100.00 µg/g. 30% of the isolates produced this toxic substance at a range of 10.00-100.00 µg/g. Based on the results, AFB_1 _was produced at a higher level in the corn and pellet samples, compared to that in the soybean meal and mash types (*P<0.05*). The mean concentrations of the three replicates of all AFs with their respective standard error bars are presented in Figure 2b.

***Evaluation of five sample assays***

In this study, 20 strains were isolated from livestock grains, out of which 5 samples were randomly selected based on the seed type and sequencing with Bt2 primer. The determination of the five strain sequences based on the GenBank revealed the types of fungi used in this study, as presented in Table 3. The Bt2 band was successful in all strains, and a total of approximately 550 pairs of bases were observed during electrophoresis.

***Phylogenetic analysis***

**Figure 3:**
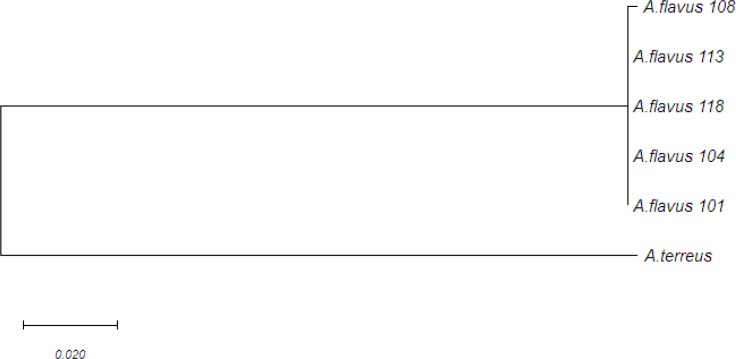
Phylogenetic tree of selected* Aspergillus flavus *isolates based on analysis of beta-tubulin gene sequences (Neighbor-joining tree model is based on the model of Tamura-Nei. Evolutionary analysis was carried out in MEGA7.)

Two separate clades in the phylogenic tree, which showed an affinity of more than 65. It was difficult to differentiate these two species given their great similarity. *Aspergillus terreus* was selected as the outgroup; however, Bt2 was able to split them well (Figure 3).

**Table 3:** Strains of *Aspergillus *section* Flavi* used in this study for sequence analysis of partial beta-tubulin 1, 4 gene

**Size of** *** BT1,4 *** **partial sequence (bp)**	**Feed**	**Strain**	**Species**
500	Corn	101	*A. flavus*
500	Corn	104	*A. flavus*
500	Pelleted feed	118	*A. flavus*
550	Soybean meal	113	*A. flavus*
550	Pelleted feed	108	*A. flavus*

## Discussion

Microflora analysis in poultry feed permits the estimation of feed deterioration and the risk of mycotoxin contamination. Aflatoxin contamination poses serious health risks for animals and humans, especially in humid environmental conditions and all types tropical climate [5, 19, 25, 26]. Based on multiple studies, *Aspergillus *is the main mycoflora of poultry feed pellets [2, 9, 14, 18, 19]. In the current study,* A. flavus* and *A. oryzae* were isolated from both poultry feed samples and their raw materials. Other species were rarely associated with agricultural products because *A. flavus* was highly abundant in the soil and plant used for the formulation of poultry ration. 

In a review study performed by Klich on 327 articles about *A. flavus*, this fungus was found in animal tissues, soil, grain, and forage in all latitudes, especially in tropical and subtropical areas. In addition, this species was reported to be toxigenic at the rate of 30-40% [27]. The thermoresistancy of the two detected isolates in the pelletizing machine indicates the potential of AFs production in poultry feed. Processing of pelleted poultry feed cannot affect the fungal conidia and variable resistance of fungal aflatoxigenic strain [27, 28]. Azarakhsh (2011) showed that *A. flavus* was the most prevalent species, followed by *A. niger* and *A. fumigatus,* in the broiler feeds in Kermanshah province in the West of Iran [7]. In the present study, the results of molecular analysis revealed that toxigenic isolates in the corn samples had the highest and lowest ability of toxin production, compared to that in the soybean meal and poultry feed. Based on these results, any isolate could produce different amounts of AFB_1_ on YES culture. The mean level of B_1_ toxin in all feedstuffs ranged from11.28±1.18 to 2239.92 ±92.26 µg/g fungal dry weight. The results of this study are consistent with those of other studies [25, 29, 30].

In a study conducted on 58 commercial poultry feed samples obtained from 17 provinces in five geographic regions in Nigeria, Ezekiel (2014) [30] reported the presence of *A. flavus* at a frequency of 91.8%, 29% of which were toxigenic with the ability to produce AFB_1_ at a maximum concentration of 441.00 µg/g. Accordingly, our results are in line with those obtained by Klich and Ezekiel. Feed level of AFs can be affected by toxin binder that is one of the inhibitors, such as organic acids/fungicide/prebiotic, which are added in it [27, 30]. The high quantity of AFB_1_ produced by the toxigenic isolates implies the potential of aflatoxicosis at the early stage of poultry growth, especially in starter feed [31, 32]. 

Toxin production capability is very important in poultry industry from an economic perspective; however, in practice, it may not be significant in the ecological study of a fungus. It seems that the relative humidity and of the feed and field can affect other factors accounting for toxin production. Toxin production ability is significantly affected by numerous environmental factors. The β-tubulin gene is selected as a marker for the identification of *Aspergillus* species [17, 26]. Because β-tubulin is the first gene isolated and sequenced from *A. flavus*. 

In a study, among 134 fungal strains isolated from 65 samples, two fungal isolates were identified as *A. oryzae* MAO103 and *A. oryzae* MAO104 by sequencing the β-tubulin gene. They were able to degrade more than 90% of AFB_1_ in a culture broth in 14 days [33]. In another study, the PCR sequencing of β-tubulin gene in clinical samples showed that the most prevalent species were *A. flavus*, *A. oryzae,* and *A. fumigatus*, respectively [26]. In a study, β-tubulin gene sequences were used to identify the *Aspergillus* section *Flavi* in chestnuts. In the mentioned research, the main species was *A. flavus*, followed by *A. oryzae* var. *effusus*, *A. tamarii*, *A. parasiticus,* and *A. toxicarius *[34].

In the present study, the identification of *Aspergillus* strains was accomplished using β-tubulin gene sequences, revealing *A. flavus* 101, *A. flavus* 104, *A. flavus* 118, *A. flavus *113, and *A. flavus*108. Phylogenetic trees were constructed with unambiguously aligned sequences using the neighbor-joining method with the Tamura-Nei parameter as a substitution model in the MEGA7 software. In this process, *A. terreus* was selected as the outgroup. The reliability of internal branches was assessed using the bootstrap method with 1,000 replicates.

The results revealed no correlation in the ability to produce AFB_1 _by *A. flavus* on YES broth and the level of toxin in the poultry feed. In this regard, the presence of* A. flavus* fungi in human food or animal feed does not mean the presence of AFs in the material because the conditions required for the growth of the fungus and AFs production are different. Probst (2011) [29] reported that the non-toxigenic isolates of *A. flavus* and presence of other fungi could affect the capacity of AF production in food/feed. Presence of AFB_1_ in feedstuffs can be dangerous to human health; therefore, it is required to adopt a monitoring system to predict the production of this toxin in poultry feed. Based on the evidence, AFB_1_ exists in a wide range of feedstuffs depending on the region and climate.

A great body of research on mycotoxins showed that 98% of the ingredients used in animal feed formulation are positive for AFs. Moreover, maize has been reported as a preferred substrate for fungal growth and mycotoxin production in comparison with soybean and wheat in poultry feed [35-38].

## Conclusion

Given the wide distribution of *A. flavus* spores in the environment, the risk of the contamination of corn and pelleted poultry feed with this species is very high as evidenced in the present study. The capacity of producing different levels of AFB_1_ in corn is a great hazard in the quality control of poultry feed. These data provide information on the toxigenic feature of *A. flavus* in feed materials and allow us to identify the sources of this contamination. Accordingly, this information should could be helpful for the adoption of safety measures in the formulation of poultry feed to prevent the incidence of subsequent disease. 

According to the molecular analysis, *A. flavus* was the isolates with the highest level of toxin production in the corn samples (No. 101 and 104) and the soybean meal samples (No. 113) had an average level of AFB_1_ production; therefore, *A. flavus *No.118 and* A. flavus* No.108 were found in the pelleted feed. Feeding is an important issue in poultry farming; therefore, it is recommended to routinely control all raw materials in feed mill factories. Unprocessed materials in poultry feed, such as corn, as well as pelleted feed, needs further monitoring for the presence of *A. flavus* and *A. oryzae* since they can be more hazardous when conditions are not optimal for destroying the fungus and the associated toxins. 
